# A review of current practices in periprosthetic joint infection debridement and revision arthroplasty

**DOI:** 10.1186/s42836-022-00136-5

**Published:** 2022-09-01

**Authors:** Humza M. Mian, Joseph G. Lyons, Joshua Perrin, Andrew W. Froehle, Anil B. Krishnamurthy

**Affiliations:** 1 Department of Orthopaedic Surgery, Wright State University Boonshoft School of Medicine, 30 E. Apple St. Suite #2200, Dayton, OH 45409 USA; 2 Wright State University Boonshoft School of Medicine, Wright State Physicians Bldg, 725 University Blvd., Dayton, OH 45435 USA; 3 School of Nursing, Kinesiology and Health, Wright State University, 3640 Colonel Glenn Hwy., Dayton, OH 45435 USA

**Keywords:** Prosthesis-related infections/prevention & control, Prosthesis-related infections/surgery, Debridement, Anti-bacterial agents, Arthroplasty, Biofilms, Knee prosthesis, Hip prosthesis, Orthopedic surgeons

## Abstract

**Background:**

Periprosthetic joint infection remains a significant challenge for arthroplasty surgeons globally. Over the last few decades, there has been much advancement in terms of treatment and diagnosis, however, the fight rages on. As management of periprosthetic joint infections continues to evolve, it is critical to reflect back on current debridement practices to establish common ground as well as identify areas for future research and improvement.

**Body:**

In order to understand the debridement techniques of periprosthetic joint infections, one must also understand how to diagnose a periprosthetic joint infection. Multiple definitions have been elucidated over the years with no single consensus established but rather sets of criteria. Once a diagnosis has been established the decision of debridement method becomes whether to proceed with single *vs* two-stage revision based on the probability of infection as well as individual patient factors. After much study, two-stage revision has emerged as the gold standard in the management of periprosthetic infections but single-stage remains prominent with further and further research.

**Conclusion:**

Despite decades of data, there is no single treatment algorithm for periprosthetic joint infections and subsequent debridement technique. Our review touches on the goals of debridement while providing a perspective as to diagnosis and the particulars of how intraoperative factors such as intraarticular irrigation can play pivotal roles in infection eradication. By providing a perspective on current debridement practices, we hope to encourage future study and debate on how to address periprosthetic joint infections best.

## Background

Periprosthetic joint infections (PJI) continue to be one of the most significant challenges facing arthroplasty surgeons today. Current data estimate the risk of PJI at approximately 2% [1–3] after primary procedure and as high as 15% [4–7] after revision procedures, which has led us to over a billion dollars in healthcare spending annually in managing PJI [8]. Our aim through this perspective is to address and review current concepts in PJI debridement. In order to understand the role debridement plays in PJI, we first discuss criteria for PJI diagnosis. As such, we will review several viewpoints regarding the role of debridement in PJI: diagnosis of PJI, role of intra-articular irrigation, debridement, and antibiotic implant retention (DAIR), one-stage revision, and the gold standard being two-stage revision. Considering the morbidity posed by PJI and the ever-increasing number of arthroplasty procedures performed annually, reviewing treatment of PJI remains as critical as ever to ensuring optimal quality of life for patients whose only goal is to minimize sometimes decades worth of arthritic pain.

## Main text

### Diagnosis of periprosthetic joint infection

In this section, we will discuss the evolution of relevant diagnostic criteria that guide orthopedic surgeons across the world in diagnosing PJI. While multiple definitions for PJI have been suggested, there remains no universally accepted definition for PJI. In 2011, the Musculoskeletal Infection Society (MSIS) convened and set forth a set of criteria to assist in diagnosing PJI. The criteria are divided into major and minor criteria respectively. The major criteria include the following: presence of a sinus tract communicating directly with the prosthesis, or a single pathogen grown on culture based on a minimum of two distinct aspirates or tissue specimens from the joint in question. Presence of either of these criteria would, according to the 2011 MSIS definition, indicate presence of a periprosthetic joint infection. In the absence of either major criterion, the society recommends the presence of at least 4 of 6 minor criteria which include the following: elevated leukocyte count, elevated serum C-reactive protein (CRP), elevated erythrocyte sedimentation rate (ESR), elevated neutrophil percentage isolated from synovial fluid, gross purulence within the suspected joint, the positive isolation of one pathogen in a single culture or sample of periprosthetic synovial fluid, or finally more than five neutrophils identified on a high-powered microscopic field from five distinct fields seen on periprosthetic tissue histology [9, 10]. These criteria were externally validated in a multicenter study published seven years later that included upwards of 1500 patients. In this cohort study, elevations in serum CRP and ESR were the two most predictive variables associated with PJI [11]. 

Two years after the MSIS definition was developed, a conference of some 400 delegates from over 50 countries convened in Philadelphia to reassess the MSIS criteria ahead of the annual MSIS meeting. This meeting came to be known as the International Consensus Meeting on Periprosthetic Joint Infection (ICM). Based on their collective wisdom and review of the available literature, the group decided to include pathologically-elevated leukocyte esterase values as an additional minor criterion [12–14]. While leukocyte esterase had been discussed earlier at the 2011 MSIS meeting, there was concern about the reliability of the test in part due to concerns regarding different reagents and testing materials. Controversy surrounding the inclusion of leukocyte esterase revolves around the confounding effect that blood can have on reading the results as documented in a prospective cohort study conducted at two centers. The study evaluated the limitations of leucocyte esterase reagent (LCR) strips. They can be deemed indecipherable due to colorimetric nature of the strip pad, which simultaneously introduces human error, by way of subjectivity, into the equation as well [15–17]. In addition, there has been significant discussion concerning other reagents playing a role in the diagnosis of PJI, such as alpha defensin or D Dimer [11, 18]. With the addition of leukocyte esterase, there was an increase in sensitivity of diagnosing PJI, with a small decrease from around 98% to 95% in specificity [17]. Additionally, PCR techniques have been suggested as possible replacements for current serological testing. While there may be a role in the future, multiple studies have demonstrated that PCR testing is not superior, in the diagnosis of PJI, to the already established criteria and can even prove inferior to culture identification. In fact, specificity of PCR-driven diagnosis techniques can be as low as 75%, with a positive predictive value under 40% [19–24].

In 2018, the MSIS conducted the most significant overhaul of the existing PJI criteria to date. They incorporated synovial alpha defensin as a significant minor criterion, which was given a score of 3 points, which was higher than the individual value given to any other minor criteria. Sensitivity and specificity were approximately 98% and over 99% respectively based on external validation, which was higher than previous scoring criteria [18, 25–27]. Later in 2018 and 2019, the definition was given another thorough review by the European Bone and Joint Infection Society (EBJIS) with support from the MSIS, European Society of Clinical Microbiology and Infectious Diseases (ESCMID). Rather than splitting the criteria into major and minor groups, the EBJIS had three distinct groups based on existing clinical and serological markers: infection likely, infection unlikely, or infection confirmed. As in previous definitions, this definition relies on clinical examinations, serological markers such as CRP, leukocyte count with PMN%, alpha defensin, culture results, sonication results, histology, as well as incorporating nuclear imaging. Up to this point, none of the previously discussed criteria incorporated sonication into the diagnosis of PJI. Within the context of PJI, sonication is the targeting of bacterial biofilms with sound waves to disrupt or dislodge pathogenic bacteria. This would, in turn, increase the likelihood of resulting positive cultures and therefore diagnosing a PJI. This technology has been externally validated at length, demonstrating its utility in diagnosis. In 2007, a large prospective trial demonstrated an almost 18% increase in sensitivity in diagnosing PJI when using sonicated periprosthetic joint tissue *vs*. without. The same trial also found the gap in sensitivity to significantly widen to 35% when evaluating patients on antibiotic therapy [21, 28]. While specificities were comparable with less than a 1% difference, the stark divide in sensitivity highlights the important role sonication can play. While this study demonstrated overwhelmingly the benefits of sonication in diagnosing PJI, there has been supporting literature indicating that using sonication on arthroplasty implants can increase the likelihood of tissue culture positivity, increasing sensitivity of PJI diagnosis [28–32]. Despite the value of sonication technology as previously mentioned, it has yet to be incorporated into any diagnostic criteria outside the recent EBJIS definition. In turn, the EBJIS diagnostic criteria have yet to be externally validated in a meaningful study. Overall, definitions and criteria continue to be revised and augmented with the study of new and emerging technologies that can help guide us toward a universally-accepted set of criteria for PJI diagnosis. Some of these new technologies aimed at addressing biofilms include the following: cathodic voltage controlled electrical stimulation, electrochemical scaffolds, hyperthermia, polyclonal antibodies, antimicrobial peptides, and endogenous molecules such as bacteriophages [33–35]. A complete discussion of each of these technologies and their respective efficacy falls outside the scope of this paper, however, it is crucial to keep molecular level treatments in mind as we focus on the macromolecular treatments offered through standard revision arthroplasty and the debridement techniques which are the focus of this paper.

### Treatment of PJI

#### Role of intra-articular irrigation

In the past decade alone, there has been much debate as to which antiseptic or antibacterial solution is superior in treating PJI. However, what is not debated is the role that intra-articular irrigation plays and its importance in treating PJI. Most can agree that the primary goal of any intra-articular irrigation solution is eradication of the biofilm that leads to such a high reinfection rate and eventually multiple revision surgeries [36, 37]. As biofilm is the primary target of any debridement technique, we should briefly review the concept itself. Biofilms have been suggested as the most critical factor influencing PJI treatment. Biofilms essentially are formed by any bacteria and occur on foreign surfaces such as implants. The threat posed by biofilms lies in their inherent ability to generate a microcosm of pathogenic growth shielded from conventional antibiotic delivery systems. Oftentimes, bacteria protected by biofilms can multiply their resistance to antibiotics upwards of 1000 times. Failure to address the biofilm ensures recurrence of infection in short order, highlighting the importance of proper debridement [38–42].

*Staphylococcus* species has long been known to cause PJI. *S. epidermidis* and is the most common offending agent and forms sturdy biofilms on implanted structures [43, 44]. *S. aureus* is another species that has been shown to be an independent risk factor for treatment failure, most likely due to its penchant for antibiotic resistance [44–47]. *S. aureus* has also been shown to be the leading cause of hematogenous total knee arthroplasty infections, which are less receptive to treatment [48, 49]. Based on these concerning findings, *S. aureus* has been the target of many studies.

Much of the current literature reviewing intra-articular irrigation focuses on *in vitro* studies. In fact, one study compared commercially-available solutions including Bactisure (Zimmer Biomet, Warsaw, IN, USA) and Irrisept (Irrimax, Gainseville, FL, USA) against diluted betadine and sodium chloride solutions. These solutions were tested against strains of *S. aureus* and *S. epidermidis*. During this *in vitro* study, media treated with Irrisept were found to have the highest statistically significant remaining colonies of all bacterial strains when compared to other solutions. Betadine and Bactisure, which is a combination of water, sodium acetate, benzalkonium chloride, acetic acid, and ethanol, were comparable in their efficacy in eradicating bacterial biofilms [50, 51].

Furthermore, studies have evaluated the role of antibiotic-supplemented solutions. For example, vancomycin combined with poviodine solutions when compared to povidone alone in a large retrospective cohort looking at over 11,000 patients did demonstrate relative reduction in infection risk but no significant difference was found when it came to PJI incidence [52]. Besides vancomycin, gentamicin has also been evaluated as an adjunct to irrigating solutions, with similar findings suggesting that antibiotic supplementation may not be as beneficial as one would imagine [53]. A polymyxin and bacitracin irrigation solution has similarly been tried and demonstrated as inferior to betadine solution in eradicating biofilms [54]. In a randomized controlled trial published in 2020, betadine solution was compared to saline lavage and there was, in fact, a difference in the number of postoperative infections. There was a significant decrease in the number of postoperative infections in the group utilizing the betadine lavage over the saline lavage [55].

The ongoing debate as to whether solutions such as betadine are superior to chlorhexidine gluconate-based solutions illustrates the useful adjunct that intra-articular irrigation plays. In choosing which solution is superior, most agree that it should be capable of penetrating the biofilm that is a hallmark of chronic PJI, while remaining minimally-cytotoxic to host tissue that will prove crucial towards the inevitable lengthy healing process [56–59]. In addition to the contents of the irrigating solution, there has been significant discussion regarding appropriate irrigation pressure and duration. Multiple studies have compared high pressure lavage *vs*. low pressure lavage, with the majority of available literature corroborating the effectiveness of low pressure lavage in favor of high pressure lavage [60–64]. When discussing duration of irrigation, an *in vitro* study demonstrated that bacteria must be exposed to a minimum of 120 seconds of irrigation using commonly available irrigation solutions such as, but not limited to, poviodine, chlorhexidine, and acetic acid based solutions [65].

#### One-stage and DAIR

One-stage revision arthroplasty or single-stage revision (SSR) has been gaining favor amongst orthopedists in recent years due to decreased mortality, comparable infection eradication rates, and lowered cost when compared to two-stage revision arthroplasty procedures [66–69]. The first step in a one-stage revision protocol is to achieve adequate exposure to the affected area. Once exposed, local excision of skin margins, synovium, and any sinus tracts is performed similar to any other debridement procedure. All implants are then removed, and then copious irrigation follows. After the surgeon has appropriately debrided, removed the implants, and then irrigated the infected joint, the new implants are then placed. Most often, the cement used during the revision procedure is impregnated with antibiotics.

Indications for a single-stage revision arthroplasty (SSR) currently remain broad and ill-defined, however, they continue to evolve. In one study, patients were eligible for one-stage revision if they had symptoms lasting longer than 4 weeks and did not have systemic symptoms, extensive soft tissue involvement that would prevent wound closure, fungal infection, HIV infection, or they were on chemotherapy [70]. In another review article comparing one- and two-stage revision arthroplasty, it was determined that relative indications for one-stage revision over two-stage were an affected THA, good soft tissue, appropriately identified organism with susceptibility to oral agents, good bone stock, and no bone grafting required [69]. These indications for one-stage revision are substantiated by a consensus article published in the *Journal of Orthopaedic Research* in 2014. In the article, it was determined that indications for one-stage revision arthroplasty included circumstances where effective antibiotics were available but did not include patients with systemic manifestations of infection [71]. In terms of the evolution of single stage revision, there has been significant advancement in recent years. One of the hallmarks of the new set of indications is the need for proper identification of the offending microbe with appropriate sensitivity and resistance testing completed prior to initial debridement. Knowing what organism is most likely responsible has significant impact on the likelihood of success of SSR and, as such, has become a major indication for SSR if this knowledge is available [72, 73]. Armed with this knowledge, the operating surgeon is able to select the appropriate antibiotic to load into the cement for the revision as well as optimize the postoperative antibiotic plan to ensure the best chance of success [74, 75]. In the past, relative indications to proceed have relied on the absence of gross signs of infection or instability such as radiographic loosening, radiographic signs of osseous inflammation, and grossly draining sinus tracts [76–79]. In one clinical study, WBC, CRP, and joint aspirate results were not associated with treatment failure. However, identification of *Streptococcus* species was conversely associated with treatment success. Additionally, elevated ESR values above approximately 47 mm/h were associated with treatment failure, suggestive of more deep-seated and chronic infections not amenable to SSR [76]. Body mass index, which has been historically associated with high risk for infection after primary arthroplasty, may have a role to play in determining success of SSR as well. While not well borne out in the literature there were some data to suggest that higher BMI is associated with failure of SSR [80]. Ultimately, outcomes in terms of treatment success for SSR have a wide range of little clinical significance stretching from less than 20% to above 80% in current literature [81–85]. Relative contraindications for one-stage revision were voted to be lack of identification of an organism preoperatively, the presence of a sinus tract, or severe soft tissue involvement that may lead to the need for flap coverage [71]. More research needs to be done in order to come to a consensus on what criteria best justify a one-stage revision.

We also discuss debridement, antibiotics, and implant retention (DAIR) in this section as it is similar in scope to a one-stage revision. DAIR is a surgical procedure that is often utilized as an alternative to one- and two-stage revision arthroplasty to treat prosthetic joint infection (PJI). When compared to revision arthroplasty, DAIR has been associated with superior functional outcomes due to its ability to minimize bone loss and soft tissue trauma [86, 87]. There is also a reduced risk of intraoperative fracture due to implant retention, and overall procedure times are shorter on average [88, 89]. Some studies even reported faster postoperative rehabilitation when compared to other revision procedures [90]. However, DAIR is not without its drawbacks. Unlike one- and two-stage revisions, DAIR protocols reported variable infection cure rates [91–93]. Additionally, the success of DAIR is questionable in scenarios such as the presence of hip fracture [94, 95]. In the following paragraphs we discuss some of the current concepts regarding debridement, antibiotics, and implant retention.

Indications to perform a DAIR procedure are not rigidly defined. It has been suggested that a short duration of infection in a non-immunocompromised host with identified and antibiotic-sensitive organisms are relative indications for a DAIR procedure [96]. Relative contraindications to DAIR include chronic infection, infection with multi-drug-resistant organisms, polymicrobial infections, and fungal organisms [97, 98]. The steps in a DAIR procedure are similar to a one-stage revision, except the implants are never removed. Modular components, such as the plastic spacer, may be removed to improve visibility of the posterior capsule or even exchanged, but the remaining structures are not removed. Because the components are left intact, it is vital to perform extensive debridement and thorough irrigation, most often with 6–9 liters of normal saline. There is increasing support for irrigating the infected joint with antiseptic solutions, such as chlorhexidine and betadine, as previously discussed. Both solutions have been shown to prevent postoperative infection after arthroplasty [55, 99] and have yielded similar results when used with revision arthroplasty [55]. Some orthopedic surgeons have adopted this practice and have been utilizing antiseptic solutions in their DAIR protocols with success [100, 101]. 

Pulse lavage is another irrigation technique that can be used with a DAIR protocol. In one study, pulsed lavage achieved a 94% 2-year survival rate free of treatment failure [102]. Despite this promising outcome, pulsed lavage has not been proven superior to standard lavage techniques in other studies [103]. Once debridement, irrigation, and exchange of modular implant components are completed, the wound is often closed and a suction drain left in place. There is also the option to outfit the patient with a continuous closed irrigation system, however, one study showed continuous irrigation achieved the same infection control as a suction drain, but with the disadvantage of increasing hospital stay time [104]. This makes a strong argument for the use of suction drains.

After surgery, either one-stage revision or DAIR, the patient then undergoes antibiotic treatment. Antibiotics are typically held preoperatively if the pathogen is unknown. In the immediate postoperative period, broad-spectrum antibiotics are started until microbiological results are obtained. Once the pathogen is identified, antibiotic therapy is initiated based on susceptibility profiles. Initially, patients are given intravenous antibiotics for 2–6 weeks following the procedure [83, 90, 92, 105]. After intravenous treatment is completed, many surgeons treat their patients with long-term oral antibiotic therapy to ensure biofilm eradication or suppression [82, 106, 107]. Some surgeons are also utilizing direct intra-articular antibiotics with some promising results [108]. 

Despite the consensus that patients who undergo a DAIR procedure require long-term antibiotics, the exact duration that they should be treated remains a point of contention. A study by Byren *et al*. in 2009 suggested that the risk of treatment failure increases 4-fold after the cessation of antibiotics, with most of those failures occurring within 4 months of antibiotic stoppage. In this study, the researchers treated their patients for an average of 1.5 years [82]. On the other hand, some studies suggest a shorter duration of antibiotics: as little as 6 weeks to as long as 6 months are just as effective at treating PJI as a more drawn-out antibiotic course [109–111]. One study even states that the only factor associated with treatment failure in their population was the antibiotic selection and not the duration of treatment [46]. The inability to determine a best practice treatment duration suggests that failure is more complex than we previously thought, and relies heavily on the infecting pathogen and how it relates to a patient’s own comorbidities. The latter has the potential to delegate a patient to lifelong antibiotic therapy, which deserves future study.

When it comes to specific antibiotic regimens used in PJI treatment, there has been a movement towards adding rifampin to existing antibiotic regimens when treating *S. aureus* infections. This addition has been shown to be superior to historical cohorts that were not treated with Rifampin [112]. Unfortunately, these results have not easily been replicated. In a randomized controlled trial, rifampin combination therapy did not have any statistically significant advantage over standard antibiotic regimens when treating *S. aureus* PJIs [113]. Becker *et al*. were able to replicate the success with improved outcomes when rifampin was added to standard therapy in their study. They found the primary determinant of treatment success to be the duration of rifampin therapy, with longer duration proving more fruitful [114]. Due to the nature of the organism, there also exist strains that are resistant to rifampin. For these cases, it is recommended to treat the patient with linezolid, as it has been associated with high remission rates and is an appropriate alternative for infections due to fluoroquinolone and/or rifampin-resistant bacteria [115]. 

As previously mentioned, the treatment success rate of DAIR is highly variable, ranging from approximately 30% to 90% efficacy [83, 116–118]. One factor not previously discussed that can determine the success of a DAIR procedure is early intervention [100]. Performing a DAIR procedure within 4 weeks of infection onset has been associated with superior outcomes than DAIR procedures performed later in the infection’s course [119]. Interestingly, one of the factors that has been associated with worse outcomes is the presence of fracture, which decreased treatment success rates [95]. Thus, in patients who have undergone hemiarthroplasty for hip fracture, it is critical to take note of the increased risk of DAIR failure. Though DAIR can have superior outcomes when compared to two-step revision arthroplasty [86], if the procedure fails, through any means, a second DAIR may be performed. However, the efficacy of the repeated procedure over a revision arthroplasty is a research topic that needs to be expanded upon.

#### Two-stage revision arthroplasty

Two-stage revision arthroplasty for PJI has long been accepted as the standard against which all other revision procedures are measured [120–123]. This mainly stems from infection cure rates being reported as high as 90% and reinfection rates around 15% [121–124]. It is also easy to overlook the advantage of being able to debride an infected joint twice and treat a patient long-term with interim antibiotics. There is also the benefit of the surgeon having a better understanding of the bony and soft tissue defects a patient may have, a benefit when planning the second surgery. This can be appreciated in the study by Hsieh *et al*. (2005), when 24 patients with massive femoral and acetabular bone loss were successfully treated with two-stage revision. They were initially treated with a temporary cement prosthesis. After a period, definitive treatment came in the form of an endoprosthesis that was cemented into an allograft designed for the bone loss [125]. 

Just like many other procedures, two-stage revisions are not without their drawbacks. The most obvious argument against this treatment protocol is the fact that the patient must undergo two separate surgeries. Perhaps most importantly, two-stage revision procedures are associated with a high mortality rate [68]. Additionally, two surgeries increase the economic burden on the hospital system. It has been estimated that the combined annual hospital cost of treating PJI with a two-stage procedure may be as high as 1.85 billion USD by 2030 [126]. There is also the concern for resource and staff allocation, especially during the COVID era when resources are often harder to come by and staff are already spread thin. Two surgeries increase the cost directly to the patient when compared to other treatment modalities. A two-stage procedure may cost anywhere from 1.5–1.7 times that of a single-stage revision [66, 67]. Compared to a DAIR protocol, two-stage revision may cost three times as much [127]. There are also hidden costs to the patient such as increased time off from work, transportation costs, increased time being disabled in the interim between surgeries, and there is always the concern for loss to follow-up.

The gap in PJI eradication between two-stage revision and other procedures is closing thanks to modern technology [87, 128]. In a retrospective cohort study assessing patient-reported outcome measures (PROMs) for single *vs*. two-stage revision of chronic infection of total hip arthroplasty, Tirumala *et al*. demonstrated a functional benefit of one-stage revision when compared to its two-stage counterpart. Both treatment modalities improved PROM scores, however, these scores were significantly higher for one-stage revision THA [129]. Two-stage revision has also undergone changes throughout the years thanks to technological advances. However, the principles of a two-stage revision surgical protocol have remained the same. In the following sections, we discuss the indications and contraindications, what a typical two-stage revision looks like as well as some of the technological advances that have shaped what the procedure has become today.

The indications for a two-stage revision arthroplasty are often not well defined. In a study assessing 253 two-stage revisions for infected TKA, the indications for two-stage surgery were evidence of chronic infection, increased CRP, positive culture report, and/or intraoperative histology consistent with infection [122]. The contraindications, in the same study, were patients with documented infection who were not able to undergo surgery or patients without evidence of infection [122]. In many cases, the indication to perform a two-stage revision is the contraindication to a one-stage one. For example, the signs and symptoms of systemic infection, inadequate tissue coverage, the presence of a sinus tract, and an unidentified infecting pathogen [71, 130]. 

A two-stage revision arthroplasty for PJI begins with surgically opening the affected joint and removing all implanted materials. The joint is then debrided of all suspicious tissues and debris. After the joint is thoroughly inspected and cultures are obtained, an antibiotic-impregnated spacer is fitted. The joint is then closed. During this interim period, the patient is typically treated with antibiotics, IV, oral, or a combination of both. Once the patient has met certain criteria, the second surgery is done, typically 6–12 weeks later. The antibiotic implant is removed, the joint is thoroughly inspected and debrided once again, and then the patient is outfitted with a new permanent prosthesis.

The antibiotic-impregnated spacer is considered one of the true advantages of a two-stage procedure. The primary goal of the spacer is to stabilize the joint and provide adequate concentrations of antibiotics to the otherwise difficult to penetrate area. Fink *et al*. (2011) wanted to know if antibiotics from a spacer could be detected in the tissues surrounding the spacer 6 weeks after implantation. They had 14 spacers, removed from 14 patients. Half of the spacers were impregnated with gentamicin and clindamycin alone; vancomycin was added to the remaining seven. They were able to detect all three antibiotics in concentrations higher than their minimum inhibitory concentration (MIC) in the surrounding tissue samples and the membrane that formed between the spacer and the native tissue [131]. These levels of antibiotic concentration for such a duration would be impossible without the use of the spacer.

The antibiotic of choice is typically determined with the help of an infectious disease specialist and pre-surgical joint aspiration microbiological studies [132]. One thing surgeons should keep in mind is the exothermic reaction that occurs when bone cement is curing. Temperatures can reach 82–86 °C (approximately 180 °F), thus it is imperative to choose a heat-stable antibiotic [133]. Once the antibiotic is chosen, the surgeon must decide whether to use a static or mobile spacer. A static spacer can simply be defined as taking up the dead space where the implant used to be and not allowing motion through the knee joint [134]. The joint may be kept in full extension or minimal flexion, at the discretion of the surgeon. There are documented advantages and disadvantages to a static spacer. The primary advantage of a static spacer is the fact that it is an effective therapy at a decreased cost to the patient [135, 136]. The disadvantages of a static spacer are increased stiffness and decreased range of motion (ROM) after the second stage procedure [137]. A meta-analysis by Guild *et al*. (2014) assessed articulating cement and static antibiotic-impregnated spacers. Through 47 studies, there were 1904 patients and 2011 knees. Of those knees, 16 were complicated by arthrodesis, with 13 of those being associated with static spacers [137]. Another meta-analysis by Ding *et al*. (2017), came to similar conclusions [138]. On the other hand, Skwara *et al*. (2016) found no significant change between preoperative and postoperative ROM in their two cohorts (articulating cement *vs*. static spacer) [139]. On the other hand, decreased ROM observed with static spacers may be a desired trait and may be useful when treating patients with ligamentous compromise or grossly unstable joints.

Articulating cement spacers are also impregnated with antibiotics, but they allow flexion and extension of the knee joint. The immediate advantage of an articulating spacer is improved postoperative ROM [137, 138, 140]. There is also evidence to suggest articulating cement spacers are better at infection eradication [137, 141]. Another possible advantage of articulating spacers is less complex re-implantation procedures. It has been demonstrated that patients with articulating procedures had less complex second surgeries, for example, tibial tubercle osteotomies, than patients with static spacers. The downside to an articulating spacer is a greater cost to the patient [135]. Thankfully, newer technologies, such as 3D printing, may be able to make articulating spacers more affordable. Kong *et al*. (2021) have shown 3D-printed articulating spacers can provide satisfactory ROM with the added benefit of mitigating bone loss [142]. There is also evidence to suggest pre-fabricated off-the-shelf articulating spacers can achieve comparable outcome measures to more personalized implants [143]. Additionally, we are beginning to see increasing data on the reinfection risk in these antibiotic articulating spacers with one study documenting survivorship free of infection after reimplantation using an antibiotic articulating spacer as over 90% at 2 years and over 85% at 5 years [144]. We can only assume these technologies will continue to be utilized in the coming years to even greater effect.

Perhaps the most controversial and least-studied area of two-stage revision procedures is the interval period. Most surgeons employ a 6–12-week interval period with antibiotics. In the beginning, patients are treated with IV antibiotics and then changed to oral medications. In a study by Hoshino *et al*. (2021), seven patients with PJI were treated with two-stage revision arthroplasty utilizing a hand-made silicone mold in the interim. Also, during the interim period, they were treated with 1 week of IV antibiotics, until laboratory values normalized (CRP, WBC, ESR). They were then treated with 3 months of oral antibiotics. Medications were chosen based on the results of microbiological studies. In this scenario, no incidence of reinfection occurred [145]. In a separate study by Burastero *et al*. (2020), 253 patients treated for PJI with two-stage revision were assessed. The reinfection rate was 4.7%. In this protocol, patients were treated with IV antibiotics routinely for 4 weeks, followed by oral antibiotics for 2 weeks, and then a washout period of 2 weeks prior to the second stage screening for infection [146]. A study by Hsieh *et al*., (2009) may help explain why both protocols, despite their differences, work. To determine the optimal duration of systemic antibiotic therapy, 99 patients with PJI who were treated with two-stage arthroplasty were split into two groups. The first group was given 4–6 weeks of antibiotic treatment. The second group was put on 1 week of antibiotic therapy. In the end, the short-term therapy was not associated with higher rates of treatment failure [147]. The results of this study suggest treatment success is not dependent on systemic antibiotic therapy during the interval period. In the most interesting case by Stockley *et al*. (2008), no systemic antibiotics were used at all. These researchers treated 114 patients for PJI using antibiotic-loaded cement with no systemic therapy during the interim. They were able to successfully eradicate infection in 87.7% of their patients [148]. The results strongly suggest that systemic antibiotics are not an essential part of a two-stage revision. Understanding important aspects of the interval period, such as how long it should be, what antibiotics should be used (if any), and when we know a patient is ready for it to be over, are all areas that further research needs to focus on. Ultimately, when it comes to outcomes, differences in infection rates may not be as dissimilar as some would think. In fact, data from one meta-analysis from the *European Journal of Epidemiology* looking at almost 100 studies to identify differences in reinfection rates between one-stage and two-stage revisions, surprisingly, pointed to no statistically significant difference in infection rates [79]. This alone suggests that further research is necessary to identify benefits of one-stage *vs*. two-stage procedures.

Over the course of this review, we have provided a perspective of PJI diagnosis and treatment, highlighting current practices. Eradication of the biofilm that develops on arthroplasty implants is one of the hallmarks in treatment and the debridement practices described above aim to do just that. Single-stage debridement can play a role in select situations as definitive management for PJI, but two-stage revision is the preferred method in more cases based on the results we have noted. Ultimately, treatment of periprosthetic joint infections will continue to evolve with the constant evolution of antibiotic resistance weighed against our future study and debridement technique evolution.

## Data Availability

Not applicable.

## Abbreviations

PJI: Periprosthetic joint infection
DAIR: Debridement antibiotics and implant retention
CRP: C reactive protein
ESR: Erythrocyte sedimentation rate
PCR: Polymerase chain reaction
MSIS: Musculoskeletal Infection Society
ICM: International Consensus Meeting on Periprosthetic Joint Infection
EBJIS: European Bone and Joint Infection Society
ESCMID: European Society of Clinical Microbiology and Infectious Diseases
LCR: Leukocyte esterase reagent strips

## References

[1] Parvizi J, Jacovides C, Zmistowski B, Jung KA (2011). Definition of periprosthetic joint infection: is there a consensus?. Clin Orthop Relat Res.

[2] Osmon DR, Berbari EF, Berendt AR, Lew D, Zimmerli W, Steckelberg JM, et al. Diagnosis and management of prosthetic joint infection: clinical practice guidelines by the Infectious Diseases Society of America. Clin Infect Dis. 2013;56(1). Available from: https://pubmed-ncbi-nlm-nih-gov.ezproxy.libraries.wright.edu/23223583/. [Cited 2022 Jan 11]. 10.1093/cid/cis803 23223583

[3] Ong KL, Kurtz SM, Lau E, Bozic KJ, Berry DJ, Parvizi J (2009). Prosthetic joint infection risk after total hip arthroplasty in the Medicare population. J Arthroplasty.

[4] Sperling JW, Kozak TKW, Hanssen AD, Cofield RH (2001). Infection after shoulder arthroplasty. Clin Orthop Relat Res.

[5] Singh JA, Sperling JW, Schleck C, Harmsen WS, Cofield RH (2012). Periprosthetic infections after total shoulder arthroplasty: a 33-year perspective. J Shoulder Elb Surg.

[6] Padegimas EM, Maltenfort M, Ramsey ML, Williams GR, Parvizi J, Namdari S (2015). Periprosthetic shoulder infection in the United States: incidence and economic burden. J Shoulder Elb Surg.

[7] Clark JJC, Abildgaard JT, Backes J, Hawkins RJ (2018). Preventing infection in shoulder surgery. J Shoulder Elb Surg.

[8] Kurtz SM, Lau E, Watson H, Schmier JK, Parvizi J (2012). Economic burden of periprosthetic joint infection in the United States. J Arthroplasty.

[9] Commons JD, Parvizi J, Gehrke T. Definition of periprosthetic joint infection. 2014. Available from: http://jdc.jefferson.edu/rothman_institute/49. [Cited 2022 Jan 11]. 10.1016/j.arth.2014.03.009 24768547

[10] Parvizi J, Zmistowski B, Berbari EF, Bauer TW, Springer BD, Della Valle CJ (2011). New definition for periprosthetic joint infection: from the Workgroup of the Musculoskeletal Infection Society. Clin Orthop Relat Res.

[11] Shahi A, Kheir MM, Tarabichi M, Hosseinzadeh HRS, Tan TL, Parvizi J (2017). Serum D-dimer test is promising for the diagnosis of periprosthetic joint infection and timing of reimplantation. J Bone Joint Surg Am.

[12] Parvizi J, Gehrke T, Chen AF (2013). Proceedings of the international consensus on periprosthetic joint infection. Bone Joint J.

[13] Shukla SK, Ward JP, Jacofsky MC, Sporer SM, Paprosky WG, Della Valle CJ (2010). Perioperative testing for persistent sepsis following resection arthroplasty of the hip for periprosthetic infection. J Arthroplasty.

[14] Schinsky MF, Della Valle CJ, Sporer SM, Paprosky WG (2008). Perioperative testing for joint infection in patients undergoing revision total hip arthroplasty. J Bone Joint Surg Am.

[15] Nguyen-Khac E, Cadranel JF, Thevenot T, Nousbaum JB (2008). Review article: the utility of reagent strips in the diagnosis of infected ascites in cirrhotic patients. Aliment Pharmacol Ther.

[16] Wetters NG, Berend KR, Lombardi AV, Morris MJ, Tucker TL, Della Valle CJ (2012). Leukocyte esterase reagent strips for the rapid diagnosis of periprosthetic joint infection. J Arthroplasty.

[17] Zmistowski B, Della Valle C, Bauer TW, Malizos KN, Alavi A, Bedair H (2014). Diagnosis of periprosthetic joint infection. J Orthop Res.

[18] Shohat N, Yacovelli S, Chisari E, Clarkson S, Mann D, Parvizi J (2021). Alpha-defensin does not provide additional benefit over leukocyte esterase in the diagnosis of periprosthetic joint infection. Expert Rev Mol Diagn.

[19] Jacovides CL, Kreft R, Adeli B, Hozack B, Ehrlich GD, Parvizi J (2012). Successful identification of pathogens by polymerase chain reaction (PCR)-based electron spray ionization time-of-flight mass spectrometry (ESI-TOF-MS) in culture-negative periprosthetic joint infection. J Bone Joint Surg Am.

[20] Rasouli MR, Harandi AA, Adeli B, Purtill JJ, Parvizi J (2012). Revision total knee arthroplasty: infection should be ruled out in all cases. J Arthroplasty.

[21] Rak M, Barlič-Maganja D, Kavčič M, Trebše R, Cőr A (2013). Comparison of molecular and culture method in diagnosis of prosthetic joint infection. FEMS Microbiol Lett.

[22] Tunney MM, Patrick S, Curran MD, Ramage G, Hanna D, Nixon JR (1999). Detection of prosthetic hip infection at revision arthroplasty by immunofluorescence microscopy and PCR amplification of the bacterial 16S rRNA gene. J Clin Microbiol.

[23] Panousis K, Grigoris P, Butcher I, Rana B, Reilly JH, Hamblen DL (2005). Poor predictive value of broad-range PCR for the detection of arthroplasty infection in 92 cases. Acta Orthop.

[24] Mariani BD, Martin DS, Levine MJ, Booth RE, Tuan RS. The Coventry Award. Polymerase chain reaction detection of bacterial infection in total knee arthroplasty. Clin Orthop Relat Res. 1996;331(331):11–22. 10.1097/00003086-199610000-00003 8895614

[25] Iannotti F, Prati P, Fidanza A, Iorio R, Ferretti A, Prieto DP (2020). Prevention of Periprosthetic Joint Infection (PJI): a clinical practice protocol in high-risk patients. Trop Med Infect Dis.

[26] Tubb CC, Polkowksi GG, Krause B (2020). Diagnosis and prevention of periprosthetic joint infections. J Am Acad Orthop Surg.

[27] Parvizi J, Tan TL, Goswami K, Higuera C, Della Valle C, Chen AF (2018). The 2018 definition of periprosthetic hip and knee infection: an evidence-based and validated criteria. J Arthroplasty.

[28] Trampuz A, Piper KE, Jacobson MJ, Hanssen AD, Unni KK, Osmon DR (2007). Sonication of removed hip and knee prostheses for diagnosis of infection. N Engl J Med.

[29] Liu K, Fu J, Yu B, Sun W, Chen J, Hao L. Meta-analysis of sonication prosthetic fluid PCR for diagnosing periprosthetic joint infection. PLoS One. 2018;13(4). Available from: /pmc/articles/PMC5922553/. [Cited 2022 Jan 26]. 10.1371/journal.pone.0196418 PMC5922553 29702663

[30] Piper KE, Jacobson MJ, Cofield RH, Sperling JW, Sanchez-Sotelo J, Osmon DR (2009). Microbiologic diagnosis of prosthetic shoulder infection by use of implant sonication. J Clin Microbiol.

[31] Rothenberg AC, Wilson AE, Hayes JP, O’Malley MJ, Klatt BA (2017). Sonication of arthroplasty implants improves accuracy of periprosthetic joint infection cultures. Clin Orthop Relat Res.

[32] Dudareva M, Barrett L, Figtree M, Scarborough M, Watanabe M, Newnham R (2018). Sonication versus tissue sampling for diagnosis of prosthetic joint and other orthopedic device-related infections. J Clin Microbiol.

[33] Visperas A, Santana D, Klika AK, Higuera-Rueda C, Piuzzi NS. Current treatments for biofilm-associated periprosthetic joint infection and new potential strategies. J Orthop Res. 2022; Available from: http://www.ncbi.nlm.nih.gov/pubmed/35437846 10.1002/jor.25345 PMC9322555 35437846

[34] Verderosa AD, Totsika M, Fairfull-Smith KE (2019). Bacterial biofilm eradication agents: a current review. Front Chem.

[35] Shoji MM, Chen AF (2020). Biofilms in periprosthetic joint infections: a review of diagnostic modalities, current treatments, and future directions. J Knee Surg.

[36] Knecht CS, Moley JP, McGrath MS, Granger JF, Stoodley P, Dusane DH (2018). Antibiotic loaded calcium sulfate bead and pulse lavage eradicates biofilms on metal implant materials in vitro. J Orthop Res.

[37] Ernest EP, Machi AS, Karolcik BA, LaSala PR, Dietz MJ (2018). Topical adjuvants incompletely remove adherent *Staphylococcus aureus* from implant materials. J Orthop Res.

[38] Gristina AG, Costerton JW (1985). Bacterial adherence to biomaterials and tissue. The significance of its role in clinical sepsis. J Bone Joint Surg Am.

[39] Donlan RM (2002). Biofilms: microbial life on surfaces. Emerg Infect Dis.

[40] Stoodley P, Kathju S, Hu FZ, Erdos G, Levenson JE, Mehta N (2005). Molecular and imaging techniques for bacterial biofilms in joint arthroplasty infections. Clin Orthop Relat Res..

[41] Yagi H, Kihara S, Mittwede PN, Maher PL, Rothenberg AC, Falcione ADCM (2021). Development of a large animal rabbit model for chronic periprosthetic joint infection. Bone Joint Res.

[42] Alkam D, Jenjaroenpun P, Ramirez AM, Beenken KE, Spencer HJ, Smeltzer MS (2021). The increased accumulation of staphylococcus aureus virulence factors is maximized in a purR mutant by the increased production of SarA and decreased production of extracellular proteases. Infect Immun.

[43] Flurin L, Greenwood-Quaintance KE, Patel R (2019). Microbiology of polymicrobial prosthetic joint infection. Diagn Microbiol Infect Dis.

[44] Li ZL, Hou YF, Zhang BQ, Chen YF, Wang Q, Wang K (2018). Identifying common pathogens in periprosthetic joint infection and testing drug-resistance rate for different antibiotics: a prospective, single center study in Beijing. Orthop Surg.

[45] Brandt CM, Duffy MCT, Berbari EF, Hanssen AD, Steckelberg JM, Osmon DR (1999). Staphylococcus aureus prosthetic joint infection treated with prosthesis removal and delayed reimplantation arthroplasty. Mayo Clin Proc.

[46] Tornero E, Morata L, Martínez-Pastor JC, Angulo S, Combalia A, Bori G (2016). Importance of selection and duration of antibiotic regimen in prosthetic joint infections treated with debridement and implant retention. J Antimicrob Chemother.

[47] Azzam KA, Seeley M, Ghanem E, Austin MS, Purtill JJ, Parvizi J (2010). Irrigation and debridement in the management of prosthetic joint infection: Traditional indications revisited. J Arthroplasty.

[48] Iza K, Foruria X, Moreta J, Uriarte I, Loroño A, Aguirre U (2019). DAIR (Debridement, Antibiotics and Implant Retention) less effective in hematogenous total knee arthroplasty infections. J Orthop Surg Res.

[49] Rodríguez D, Pigrau C, Euba G, Cobo J, García-Lechuz J, Palomino J (2010). Acute haematogenous prosthetic joint infection: prospective evaluation of medical and surgical management. Clin Microbiol Infect.

[50] Kia C, Cusano A, Messina J, Muench LN, Chadayammuri V, Mccarthy MB, et al. Effectiveness of topical adjuvants in reducing biofilm formation on orthopedic implants: an *in vitro* analysis. Available from: 10.1016/j.jse.2020.12.009. [Cited 2021 Nov 17]. 10.1016/j.jse.2020.12.009 33529773

[51] O’Donnell JA, Wu M, Cochrane NH, Belay E, Myntti MF, James GA (2021). Efficacy of common antiseptic solutions against clinically relevant microorganisms in biofilm. Bone Joint J.

[52] Iorio R, Yu S, Anoushiravani AA, Riesgo AM, Park B, Vigdorchik J (2020). Vancomycin powder and dilute povidone-iodine lavage for infection prophylaxis in high-risk total joint arthroplasty. J Arthroplasty.

[53] Yazdi H, Moradi A, Herbort M (2014). The effect of gentamicin in irrigating solutions on articular infection prophylaxis during arthroscopic ACL reconstruction. Arch Orthop Trauma Surg.

[54] Goswami K, Cho J, Foltz C, Manrique J, Tan TL, Fillingham Y (2019). Polymyxin and bacitracin in the irrigation solution provide no benefit for bacterial killing in vitro. J Bone Jt Surg - Am.

[55] Calkins TE, Culvern C, Nam D, Gerlinger TL, Levine BR, Sporer SM (2020). Dilute betadine lavage reduces the risk of acute postoperative periprosthetic joint infection in aseptic revision total knee and hip arthroplasty: a randomized controlled trial. J Arthroplasty.

[56] O’Donnell JA, Wu M, Cochrane NH, Belay E, Myntti MF, James GA (2021). Efficacy of common antiseptic solutions against clinically relevant microorganisms in biofilm. Bone Joint J.

[57] Böhle S, Röhner E, Zippelius T, Jacob B, Matziolis G, Rohe S. Cytotoxic effect of sodium hypochlorite (Lavanox 0.08%) and chlorhexidine gluconate (Irrisept 0.05%) on human osteoblasts. Eur J Orthop Surg Traumatol. 2021. Available from: https://pubmed-ncbi-nlm-nih-gov.ezproxy.libraries.wright.edu/33738603/. [Cited 2021 Nov 16]. 10.1007/s00590-021-02907-3 PMC8741695 33738603

[58] Goswami K, Cho J, Foltz C, Manrique J, Tan TL, Fillingham Y (2019). Polymyxin and bacitracin in the irrigation solution provide no benefit for bacterial killing in vitro. J Bone Jt Surg - Am.

[59] Severing AL, Rembe JD, Koester V, Stuermer EK (2019). Safety and efficacy profiles of different commercial sodium hypochlorite/hypochlorous acid solutions (NaClO/HClO): antimicrobial efficacy, cytotoxic impact and physicochemical parameters in vitro. J Antimicrob Chemother.

[60] Sousa R, Abreu MA (2018). Treatment of prosthetic joint infection with debridement, antibiotics and irrigation with implant retention - a narrative review. J Bone Jt Infect.

[61] Kalteis T, Lehn N, Schröder HJ, Schubert T, Zysk S, Handel M (2005). Contaminant seeding in bone by different irrigation methods: an experimental study. J Orthop Trauma.

[62] Adili A, Bhandari M, Schemitsch EH (2002). The biomechanical effect of high-pressure irrigation on diaphyseal fracture healing in vivo. J Orthop Trauma.

[63] Bahrs C, Schnabel M, Frank T, Zapf C, Mutters R, Von Garrel T (2003). Lavage of contaminated surfaces: an in vitro evaluation of the effectiveness of different systems. J Surg Res.

[64] Mundy LR, Gage MJ, Yoon RS, Liporace FA (2017). Comparing the speed of irrigation between pulsatile lavage versus gravity irrigation: an Ex-vivo experimental investigation. Patient Saf Surg.

[65] Christopher ZK, Tran CP, Vernon BL, Spangehl MJ. What is the duration of irrigation? An *in vitro* study of the minimum exposure time to eradicate bacteria with irrigation solutions. 2021. Available from: 10.1016/j.arth.2021.10.013. [Cited 2021 Nov 17]. 10.1016/j.arth.2021.10.013 34740788

[66] Klouche S, Sariali E, Mamoudy P (2010). Total hip arthroplasty revision due to infection: a cost analysis approach. Orthop Traumatol Surg Res.

[67] Merollini KMD, Crawford RW, Graves N (2013). Surgical treatment approaches and reimbursement costs of surgical site infections post hip arthroplasty in Australia: a retrospective analysis. BMC Health Serv Res.

[68] Berend KR, Lombardi AV, Morris MJ, Bergeson AG, Adams JB, Sneller MA (2013). Two-stage treatment of hip periprosthetic joint infection is associated with a high rate of infection control but high mortality hip. Clin Orthop Relat Res.

[69] Pangaud C, Ollivier M, Argenson JN (2019). Outcome of single-stage versus two-stage exchange for revision knee arthroplasty for chronic periprosthetic infection. EFORT Open Rev.

[70] Mock BK, Fehring TK (2019). One-stage exchange revision arthroplasty for the treatment of prosthetic joint infection: rational and technique. Oper Tech Orthop.

[71] Lichstein P, Gehrke T, Lombardi A, Romano C, Stockley I, Babis G (2014). One-stage versus two-stage exchange. J Orthop Res.

[72] Ji B, Wahafu T, Li G, Zhang X, Wang Y, Momin M (2019). Single-stage treatment of chronically infected total hip arthroplasty with cementless reconstruction: results in 126 patients with broad inclusion criteria. Bone Joint J.

[73] Whiteside LA, Roy ME (2017). One-stage revision with catheter infusion of intraarticular antibiotics successfully treats infected THA. Clin Orthop Relat Res.

[74] Gehrke T, Zahar A, Kendoff D (2013). One-stage exchange: it all began here. Bone Joint J.

[75] Lum ZC, Holland CT, Meehan JP (2020). Systematic review of single stage revision for prosthetic joint infection. World J Orthop.

[76] Klare CM, Fortney TA, Kahng PW, Cox AP, Keeney BJ, Moschetti WE (2018). Prognostic factors for success after irrigation and debridement with modular component exchange for infected total knee arthroplasty. J Arthroplasty.

[77] Silva M, Tharani R, Schmalzried TP. Results of direct exchange or debridement of the infected total knee arthroplasty. Clin Orthop Relat Res. 2002;(404):125–31. Available from: http://www.ncbi.nlm.nih.gov/pubmed/12439250 10.1097/00003086-200211000-00022 12439250

[78] Trebse R, Pisot V, Trampuz A (2005). Treatment of infected retained implants. J Bone Joint Surg Br.

[79] Kunutsor SK, Whitehouse MR, Blom AW, Board T, Kay P, Mike Wroblewski B, et al. One-and two-stage surgical revision of peri-prosthetic joint infection of the hip: a pooled individual participant data analysis of 44 cohort studies The Global Infection Orthopaedic Management Collaboration. Eur J Epidemiol;33. Available from: 10.1007/s10654-018-0377-9.[Cited 2022 Jan 11]. 10.1007/s10654-018-0377-9 PMC6153557 29623671

[80] Triantafyllopoulos GK, Poultsides LA, Sakellariou VI, Zhang W, Sculco PK, Ma Y (2015). Irrigation and debridement for periprosthetic infections of the hip and factors determining outcome. Int Orthop.

[81] Buller LT, Sabry FY, Easton RW, Klika AK, Barsoum WK (2012). The preoperative prediction of success following irrigation and debridement with polyethylene exchange for hip and knee prosthetic joint infections. J Arthroplasty.

[82] Byren I, Bejon P, Atkins BL, Angus B, Masters S, McLardy-Smith P (2009). One hundred and twelve infected arthroplasties treated with “DAIR” (debridement, antibiotics and implant retention): antibiotic duration and outcome. J Antimicrob Chemother.

[83] Koyonos L, Zmistowski B, Della Valle CJ, Parvizi J (2011). Infection control rate of irrigation and Débridement for periprosthetic joint infection. Clin Orthop Relat Res.

[84] Odum SM, Fehring TK, Lombardi AV, Zmistowski BM, Brown NM, Luna JT (2011). Irrigation and debridement for periprosthetic infections: does the organism matter?. J Arthroplasty.

[85] Duque AF, Post ZD, Lutz RW, Orozco FR, Pulido SH, Ong AC (2017). Is There still a role for irrigation and debridement with liner exchange in acute periprosthetic total knee infection?. J Arthroplasty.

[86] Grammatopoulos G, Bolduc ME, Atkins BL, Kendrick BJL, McLardy-Smith P, Murray DW (2017). Functional outcome of debridement, antibiotics and implant retention in periprosthetic joint infection involving the hip. Bone Jt J.

[87] Dzaja I, Howard J, Somerville L, Lanting B (2015). Functional outcomes of acutely infected knee arthroplasty: a comparison of different surgical treatment options. Can J Surg.

[88] Lamb JN, Matharu GS, Redmond A, Judge A, West RM, Pandit HG (2019). Risk factors for intraoperative periprosthetic femoral fractures during primary total hip arthroplasty. An analysis from the National Joint Registry for England and Wales and the isle of man. J Arthroplasty.

[89] Choi HR, Von Knoch F, Zurakowski D, Nelson SB, Malchau H (2011). Can implant retention be recommended for treatment of infected TKA?. Clin Orthop Relat Res.

[90] Fisman DN, Reilly DT, Karchmer AW, Goldie SJ (2001). Clinical effectiveness and cost-effectiveness of 2 management strategies for infected total hip arthroplasty in the elderly. Clin Infect Dis.

[91] Zhang CF, He L, Fang XY, Huang ZD, Bai GC, Li WB (2020). Debridement, antibiotics, and implant retention for acute periprosthetic joint infection. Orthop Surg.

[92] Deijkers RL, Elzakker, Pijls BG. Joint infection following primary THA. J Bone Joint Surg Am. 2020;5(2):1–6. 10.2106/JBJS.OA.19.00062 PMC7418914 33123664

[93] Fehring TK, Odum SM, Berend KR, Jiranek WA, Parvizi J, Bozic KJ (2013). Failure of irrigation and débridement for early postoperative periprosthetic infection knee. Clin Orthop Relat Res.

[94] Bergkvist M, Mukka SS, Johansson L, Ahl TE, Sayed-Noor AS, Sköldenberg OG (2016). Debridement, antibiotics and implant retention in early periprosthetic joint infection. HIP Int.

[95] Yassin M, Sharma V, Butt F, Iyer S, Tayton E (2020). Early peri-prosthetic joint infection after hemiarthroplasty for hip fracture: outcomes of debridement, antibiotics, and implant retention. Surg Infect (Larchmt).

[96] Zaruta DA, Qiu B, Liu AY, Ricciardi BF (2018). Indications and guidelines for debridement and implant retention for periprosthetic hip and knee infection. Curr Rev Musculoskelet Med.

[97] Kunutsor SK, Beswick AD, Whitehouse MR, Wylde V, Blom AW (2018). Debridement, antibiotics and implant retention for periprosthetic joint infections: a systematic review and meta-analysis of treatment outcomes. J Infect.

[98] Triantafyllopoulos GK, Soranoglou V, Poultsides LA, Memtsoudis SG (2016). Implant retention after acute and hematogenous periprosthetic hip and knee infections: whom, when and how?. World J Orthop.

[99] Goswami K, Austin MS (2019). Intraoperative povidone-iodine irrigation for infection prevention. Arthroplast Today.

[100] Barros LH, Barbosa TA, Esteves J, Abreu M, Soares D, Sousa R (2019). Early Debridement, antibiotics and implant retention (DAIR) in patients with suspected acute infection after hip or knee arthroplasty - safe, effective and without negative functional impact. J Bone Jt Infect.

[101] Choo KJ, Austin M, Parvizi J (2019). Irrigation and debridement, modular exchange, and implant retention for acute periprosthetic infection after total knee arthroplasty. JBJS Essent Surg Tech.

[102] Aboltins CA, Dowsey MM, Buising KL, Peel TN, Daffy JR, Choong PFM (2011). Gram-negative prosthetic joint infection treated with debridement, prosthesis retention and antibiotic regimens including a fluoroquinolone. Clin Microbiol Infect.

[103] Urish KL, DeMuth PW, Craft DW, Haider H, Davis CM (2014). Pulse lavage is inadequate at removal of biofilm from the surface of total knee arthroplasty materials. J Arthroplasty.

[104] Royo A, Bertrand ML, Ramos L, Fernandez-Gordillo F, Guerado E (2013). Is there still a place for continuous closed irrigation in the management of periprosthetic total knee infection?. Open Orthop J.

[105] Marculescu CE, Berbari EF, Hanssen AD, Steckelberg JM, Harmsen SW, Mandrekar JN (2006). Outcome of prosthetic joint infections treated with debridement and retention of components. Clin Infect Dis.

[106] Leijtens B, Elbers JBW, Sturm PD, Kullberg BJ, Schreurs BW (2017). Clindamycin-rifampin combination therapy for staphylococcal periprosthetic joint infections: a retrospective observational study. BMC Infect Dis.

[107] Lora-Tamayo J, Euba G, Cobo J, Horcajada JP, Soriano A, Sandoval E (2016). Short- versus long-duration levofloxacin plus rifampicin for acute staphylococcal prosthetic joint infection managed with implant retention: a randomised clinical trial. Int J Antimicrob Agents.

[108] Chaiyakit P, Meknavin S, Hongku N, Onklin I (2021). Debridement, antibiotics, and implant retention combined with direct intra-articular antibiotic infusion in patients with acute hematogenous periprosthetic joint infection of the knee. BMC Musculoskelet Disord.

[109] Bernard L, Legout L, Zürcher-Pfund L, Stern R, Rohner P, Peter R (2010). Six weeks of antibiotic treatment is sufficient following surgery for septic arthroplasty. J Infect.

[110] Laffer RR, Graber P, Ochsner PE, Zimmerli W (2006). Outcome of prosthetic knee-associated infection: evaluation of 40 consecutive episodes at a single centre. Clin Microbiol Infect.

[111] Puhto AP, Puhto T, Syrjala H (2012). Short-course antibiotics for prosthetic joint infections treated with prosthesis retention. Clin Microbiol Infect.

[112] El Helou OC, Berbari EF, Lahr BD, Eckel-Passow JE, Razonable RR, Sia IG (2010). Efficacy and safety of rifampin containing regimen for staphylococcal prosthetic joint infections treated with debridement and retention. Eur J Clin Microbiol Infect Dis.

[113] Karlsen ØE, Borgen P, Bragnes B, Figved W, Grøgaard B, Rydinge J (2020). Rifampin combination therapy in staphylococcal prosthetic joint infections: a randomized controlled trial. J Orthop Surg Res.

[114] Becker A, Kreitmann L, Triffaut-Fillit C, Valour F, Mabrut E, Forestier E (2020). Duration of rifampin therapy is a key determinant of improved outcomes in early-onset acute prosthetic joint infection due to Staphylococcus treated with a debridement, antibiotics and implant retention (DAIR): a retrospective multicenter study in France. J Bone Jt Infect.

[115] Morata L, Senneville E, Bernard L, Nguyen S, Buzelé R, Druon J (2014). A Retrospective Review of the Clinical Experience of Linezolid with or Without Rifampicin in Prosthetic Joint Infections Treated with Debridement and Implant Retention. Infect Dis Ther.

[116] Uriarte I, Moreta J, Mosquera J, Legarreta MJ, Aguirre U, Martínez de los Mozos JL (2019). Debridement, antibiotics and implant retention for early periprosthetic infections of the hip: outcomes and influencing factors. Hip Pelvis.

[117] Tatarelli P, Romani T, Santoro V, Spezia M, Gallo A, Ripamonti G (2021). Debridement, antibiotics and implant retention (DAIR): an effective treatment option for early prosthetic joint infections. J Infect Chemother.

[118] Sendi P, Lötscher PO, Kessler B, Graber P, Zimmerli W, Clauss M (2017). Debridement and implant retention in the management of hip periprosthetic joint infection. Bone Jt J.

[119] Boyer B, Cazorla C (2021). Methods and probability of success after early revision of prosthetic joint infections with debridement, antibiotics and implant retention. Orthop Traumatol Surg Res.

[120] Insall JN, Thompson FM, Brause BD (1983). Two-stage reimplantation for the salvage of infected total knee arthroplasty. J Bone Jt Surg Am.

[121] Pignatti G, Nitta S, Rani N, Dallari D, Sabbioni G, Stagni C (2010). Two stage hip revision in periprosthetic Infection: results of 41 cases. Open Orthop J.

[122] Mahmud T, Lyons MC, Naudie DD, MacDonald SJ, McCalden RW (2012). Assessing the gold standard: a review of 253 two-stage revisions for infected TKA knee. Clin Orthop Relat Res.

[123] Vielgut I, Schwantzer G, Leithner A, Sadoghi P, Berzins U, Glehr M (2021). Successful two-stage exchange arthroplasty for periprosthetic infection following total knee arthroplasty: the impact of timing on eradication of infection. Int J Med Sci.

[124] Goud AL, Harlianto NI, Ezzafzafi S, Veltman ES, Bekkers JEJ, van der Wal BCH. Reinfection rates after one- and two-stage revision surgery for hip and knee arthroplasty: a systematic review and meta-analysis. Arch Orthop Trauma Surg. 2021;(0123456789). Available from: 10.1007/s00402-021-04190-7 10.1007/s00402-021-04190-7 PMC9925475 34595545

[125] Hsieh PH, Shih CH, Chang YH, Lee MS, Yang WE, Shih HN (2005). Treatment of deep infection of the hip associated with massive bone loss. J Bone Jt Surg - Ser B.

[126] Premkumar A, Kolin DA, Farley KX, Wilson JM, McLawhorn AS, Cross MB (2021). Projected economic burden of periprosthetic joint infection of the hip and knee in the United States. J Arthroplasty.

[127] Merollini KMD, Crawford RW, Graves N (2013). Surgical treatment approaches and reimbursement costs of surgical site infections post hip arthroplasty in Australia: a retrospective analysis. BMC Health Serv Res.

[128] Razii N, Clutton JM, Kakar R, Morgan-Jones R (2021). Single-stage revision for the infected total knee arthroplasty. Bone Jt Open.

[129] Tirumala V, Klemt C, van den Kieboom J, Xiong L, Kwon YM (2021). Comparison of patient reported outcome measures after single versus two-stage revision for chronic infection of total hip arthroplasty: a retrospective propensity score matched cohort study. Arch Orthop Trauma Surg.

[130] Franceschini M, Pedretti L, Cerbone V, Sandiford NA (2022). Two stage revision: indications, techniques and results. Ann Jt.

[131] Fink B, Vogt S, Reinsch M, Büchner H (2011). Sufficient release of antibiotic by a spacer 6 weeks after implantation in two-stage revision of infected hip prostheses. Clin Orthop Relat Res.

[132] Fink B, Grossmann A, Fuerst M, Schäfer P, Frommelt L (2009). Two-stage cementless revision of infected hip endoprostheses. Clin Orthop Relat Res.

[133] Vaishya R, Chauhan M, Vaish A (2013). Bone cement. J Clin Orthop Trauma.

[134] Mazzucchelli L, Rosso F, Marmotti A, Bonasia DE, Bruzzone M, Rossi R (2015). The use of spacers (static and mobile) in infection knee arthroplasty. Curr Rev Musculoskelet Med.

[135] Garg P, Ranjan R, Bandyopadhyay U, Chouksey S, Mitra SR, Gupta SK (2011). Antibiotic-impregnated articulating cement spacer for infected total knee arthroplasty. Indian J Orthop.

[136] Faschingbauer M, Bieger R, Reichel H, Weiner C, Kappe T (2016). Complications associated with 133 static, antibiotic-laden spacers after TKA. Knee Surg Sport Traumatol Arthrosc.

[137] Guild GN, Wu B, Scuderi GR (2014). Articulating vs. static antibiotic impregnated spacers in revision total knee arthroplasty for sepsis a systematic review. J Arthroplasty.

[138] Ding H, Yao J, Chang W, Liu F (2017). Comparison of the efficacy of static versus articular spacers in two-stage revision surgery for the treatment of infection following total knee arthroplasty: a meta-analysis. J Orthop Surg Res.

[139] Skwara A, Tibesku C, Paletta RJR, Sommer C, Krödel A, Lahner M (2016). Articulating spacers compared to fixed spacers for the treatment of infected knee arthroplasty: a follow-up of 37 cases. Technol Heal Care.

[140] Hsu YC, Cheng HC, Ng TP, Chiu KY (2007). Antibiotic-loaded cement articulating spacer for 2-stage reimplantation in infected total knee arthroplasty. A simple and economic method. J Arthroplasty.

[141] Veltman ES, Moojen DJF, Poolman RW (2020). Improved patient reported outcomes with functional articulating spacers in two-stage revision of the infected hip. World J Orthop.

[142] Kong L, Mei J, Ge W, Jin X, Chen X, Zhang X (2021). Application of 3D printing-assisted articulating spacer in two-stage revision surgery for periprosthetic infection after total knee arthroplasty: a retrospective observational study. Biomed Res Int.

[143] Warth LC, Hadley CJ, Grossman EL (2020). Two-stage treatment for total knee arthroplasty infection utilizing an articulating prefabricated antibiotic spacer. J Arthroplasty.

[144] Chalmers BP, Mabry TM, Abdel MP, Berry DJ, Hanssen AD, Perry KI (2018). Two-stage revision total hip arthroplasty with a specific articulating antibiotic spacer design: reliable periprosthetic joint infection eradication and functional improvement. J Arthroplasty.

[145] Hoshino T, Watanabe T, Nakagawa Y, Katagiri H, Ozeki N, Ohara T (2021). Clinical outcomes of two-stage revision total knee arthroplasty in infected cases with antibiotic-loaded cement spacers produced using a handmade silicone mold. Knee Surg Relat Res.

[146] Burastero G, Alessio-Mazzola M, Cavagnaro L, Chiarlone F, Carrega G, Capello AG (2020). Conservative two-stage revision with primary components of infected total hip arthroplasty: an analysis of survival, clinical and radiographic outcomes. PLoS One.

[147] Hsieh PH, Huang KC, Lee PC, Lee MS (2009). Two-stage revision of infected hip arthroplasty using an antibiotic-loaded spacer: retrospective comparison between short-term and prolonged antibiotic therapy. J Antimicrob Chemother.

[148] Stockley I, Mockford BJ, Hoad-Reddick A, Norman P (2008). The use of two-stage exchange arthroplasty with depot antibiotics in the absence of long-term antibiotic therapy in infected total hip replacement. J Bone Jt Surg - Ser B.

